# Evaluation of mycotoxins, mycobiota and toxigenic fungi in the traditional medicine *Radix Dipsaci*

**DOI:** 10.3389/fmicb.2024.1454683

**Published:** 2024-09-20

**Authors:** Min Hu, Lulu Wang, Dapeng Su, Qingsong Yuan, Chenghong Xiao, Lanping Guo, Meidan Wang, Chuanzhi Kang, Jinqiang Zhang, Tao Zhou

**Affiliations:** ^1^Guizhou University of Traditional Chinese Medicine, Guiyang, China; ^2^State Key Laboratory of Dao-di Herbs, Beijng, China; ^3^Faculty of Biology, University of Freiburg, Freiburg, Germany

**Keywords:** traditional Chinese medicinal herbs, *Radix Dipsaci*, toxigenic fungi, mycotoxins, aflatoxins

## Abstract

Medicinal herbs have been increasingly used for therapeutic purposes against a diverse range of human diseases worldwide. However, inevitable contaminants, including mycotoxins, in medicinal herbs can cause serious problems for humans despite their health benefits. The increasing consumption of medicinal plants has made their use a public health problem due to the lack of effective surveillance of the use, efficacy, toxicity, and quality of these natural products. *Radix Dipsaci* is commonly utilized in traditional Chinese medicine and is susceptible to contamination with mycotoxins. Here, we evaluated the mycotoxins, mycobiota and toxigenic fungi in the traditional medicine *Radix Dipsaci*. A total of 28 out of 63 *Radix Dipsaci* sample batches (44.4%) were found to contain mycotoxins. Among the positive samples, the contamination levels of AFB_1_, AFG_1_, AFG_2_, and OTA in the positive samples ranged from 0.52 to 32.13 μg/kg, 5.14 to 20.05 μg/kg, 1.52 to 2.33 μg/kg, and 1.81 to 19.43 μg/kg respectively, while the concentrations of ZEN and T-2 were found to range from 2.85 to 6.33 μg/kg and from 2.03 to 2.53 μg/kg, respectively. More than 60% of the contaminated samples were combined with multiple mycotoxins. Fungal diversity and community were altered in the *Radix Dipsaci* contaminated with various mycotoxins. The abundance of *Aspergillus* and *Fusarium* increased in the *Radix Dipsaci* contaminated with aflatoxins (AFs) and ZEN. A total of 95 strains of potentially toxigenic fungi were isolated from the *Radix Dipsaci* samples contaminated with mycotoxins, predominantly comprising *Aspergillus* (73.7%), *Fusarium* (20.0%), and *Penicillium* (6.3%). Through morphological identification, molecular identification, mycotoxin synthase gene identification and toxin production verification, we confirmed that AFB_1_ and AFG_1_ primarily derive from *Aspergillus flavus*, OTA primarily derives from *Aspergillus westerdijkiae*, ZEN primarily derives from *Fusarium oxysporum*, and T-2 primarily derives from *Fusarium graminearum* in *Radix Dipsaci*. These data can facilitate our comprehension of prevalent toxigenic fungal species and contamination levels in Chinese herbal medicine, thereby aiding the establishment of effective strategies for prevention, control, and degradation to mitigate the presence of fungi and mycotoxins in Chinese herbal medicine.

## Highlights

- *Radix Dipsaci* is susceptible to contamination by a combination of AFB_1_, AFG_1_, AFG_2_, OTA, ZEN and T-2.
- Fungal diversity and community were altered in the *Radix Dipsaci* contaminated with different mycotoxins.
- The main sources of mycotoxins in *Radix Dipsaci* are *Aspergillus flavus*, *Aspergillus westerdijkiae*, *Fusarium oxysporum*, and *Fusarium graminearum*.

## 1 Introduction

Medicinal herbs have been increasingly used for therapeutic purposes against a diverse range of human diseases worldwide. Reports suggest that an estimated 80% of the global populace continues to depend on botanical medicines (Sen and Samanta, 2015); furthermore, a significant majority of pharmaceuticals originate from plant materials or are synthesized from plant-derived compounds (Markov et al., 2017; England et al., 2023). However, the presence of inevitable contaminants, such as mycotoxins, within medicinal herbs can pose significant health risks to humans, notwithstanding their therapeutic advantages. Throughout the processes of harvesting, handling, storage, and distribution, medicinal plants are vulnerable to contamination by diverse fungi, potentially leading to spoilage and the generation of mycotoxin (Ashiq et al., 2014). The escalating utilization of medicinal plants could precipitate an augmented intake of mycotoxins; hence, the contamination of these plants with mycotoxins poses a threat to human health.

Owing to the complex cultivation processes and specialized processing requirements, traditional Chinese medicinal herbs are particularly susceptible to contamination by toxigenic fungi during their production phase (Long et al., 2021). Traditional Chinese herbal medicine is mainly contaminated by toxigenic fungi such as *Aspergillus*, *Fusarium*, and *Penicillium* (Han et al., 2012). According to the relevant studies, *Aspergillus* can produce AFs including AFB_1_, AFB_2_, AFG_1_, and AFG_2_, along with ochratoxins (Abrar et al., 2013; Schrenk et al., 2020). *Fusarium* species are recognized for synthesize zearalenone (ZEN), T-2, deoxynivalenol, and fumonisins (Gavrilova et al., 2023; Marin et al., 2013; Stockmann-Juvala and Savolainen, 2008; Yao and Long, 2020). The *Penicillium* is capable of synthesizing citrinin (Perrone and Susca, 2017; Rundberget et al., 2004). The mycotoxins mentioned often exhibit carcinogenic, teratogenic, mutagenic, hepatorenal toxicity, immunotoxicity, neurotoxicity and other highly significant toxicological effects (Fang et al., 2022; Liew and Sabran, 2022). The survey reveals the pervasive presence of mycotoxin contamination, with approximately 25% of global agricultural products being annually subjected to such contamination (Moretti et al., 2017). The safety of Chinese herbal medicine has garnered significant attention, making the prevention and control of mycotoxin pollution a focal point in current research. The accidental consumption of traditional Chinese medicinal herbs contaminated with mycotoxins poses health risks (Singh et al., 2008). The improvement in living quality has led to an increased focus on health, consequently drawing attention to the issue of mycotoxin pollution in Chinese herbal medicine. Identifying the source of mycotoxin contamination in Chinese herbal medicine is a critical initial step towards ensuring the safety of these medicinal herbs.

Root herbs, owing to their direct contact with soil, exhibit a heightened susceptibility to infections by toxigenic fungi relative to other herbal categories. Additionally, root herbs, characterized by their dense texture, demonstrate resistance to the eradication of fungal spores via conventional processing techniques (Chen et al., 2013). Under favorable environmental conditions, mycotoxins synthesized by toxigenic fungi can accumulate in Chinese herbs. *Radix Dipsaci*, is derived from the dried root of *Dipsacus asper* Wall.ex Henry (Wu et al., 2022), is renowned for its liver and kidney tonifying properties, muscle and bone strengthening effects, fracture healing abilities, and its use in treating osteoporosis (Du et al., 2023; Tao et al., 2020). The processing of *Radix Dipsaci*, categorized as a medicinal root herb, necessitates undergoing a “sweating” treatment (Tao et al., 2020). However, the high temperature and humidity environment provided during this process can easily promote the growth of toxic fungi. Currently, research into the detection of mycotoxins and the identification of toxigenic fungi within *Radix Dipsaci* remains profoundly uncharted.

Studies have found that *Radix Dipsaci* can improve osteoporosis by promoting osteogenic differentiation of bone marrow stromal cells, increasing bone mineral density and changing bone histomorphology (Ke et al., 2016). In recent years, several researchers have discovered that *Radix Dipsaci* has the potential to inhibit neuronal apoptosis in the brains of patients with Alzheimer’s disease and enhance their cognitive function (Gao et al., 2018). Yang et al. (2024) discovered that contamination of *Radix Dipsaci* with AFB1 resulted in a diminished capacity to enhance mineral density and mineral salt content in the bones of diseased mice, while also impeding the generation of newborn neurons in the hippocampus, consequently leading to cognitive decline. The pharmacodynamic effects of *Radix Dipsaci* significantly compromised by the presence of mycotoxin contamination. However, current research on *Radix Dipsaci* primarily focuses on processing, chemical composition, pharmacological effects, and other related aspects. There are limited reports regarding the impact of mycotoxin contamination on *Radix Dipsaci* quality. In this study, we conducted systematic surveillance of randomly selected *Radix Dipsaci* samples to identify those contaminated with mycotoxins. To elucidate the potential impacts of mycotoxins on *Radix Dipsaci*’s fungal community, we assessed alterations in fungal community following mycotoxin contamination. By employing morphological and molecular identification techniques, alongside mycotoxin synthase gene analysis and toxin production verification, we delineated the types and levels of mycotoxins produced by each toxigenic fungus. Our results provide valuable insights that could guide the development of strategies for the prevention, control, and remediation of contaminants, ultimately ensuring the safety of the traditional medicinal herb, *Radix Dipsaci*.

## 2 Materials and methods

### 2.1 *Radix Dipsaci* sampling and detection of mycotoxins

The *Radix Dipsaci* samples were purchased from Guiyang Taisheng medicinal material market, Guangxi Yulin herbal medicine market, Anguo herbal medicine market of Hebei Province, Bozhou herbal medicine market of Anhui Province, Hehuachi herbal medicine market of Chengdu Sichuan Province, and camphor tree herbal medicine market of Jiangxi Province from September to December 2020. We collected 9–12 batchs of *Radix Dipsaci* samples in each herbal medicine market using a five-point sampling method, yielding a total of 63 batchs *Radix Dipsaci* samples. A minimum of 5 kg was procured for each batch.

The test substance and reference substance were prepared according to the method described by Ge et al. (2024): the *Radix Dipsaci* sample was extracted with acetonitrile/water/acetic acid (80/19/1, v/v/v), and purified material was added to remove impurities, evaporated to near dryness under nitroge, and finally redissolved with acetonitrile (50% v/v) to obtain the test substance solution. Standards for AFB_1_ (lot No. MSS1003), AFG_1_ (lot No. MSS1005), AFG_2_ (lot No. MSS1006), OTA (lot No. MSS1020), ZEN (lot No. MSS1024), and T-2 (lot No. MSS1023) were sourced from Qingdao Puribang Co., Ltd. (Qingdao, China). The reference substance was prepared with 50% acetonitrile solution, and finally 400 ng/mL mixed standard substance of AFB_1_, AFG_1_, AFG_2_, OTA, T-2 and 500 ng/mL ZEN was obtained.

Mycotoxin detection in *Radix Dipsaci* utilized high performance liquid chromatography mass spectrometry (HPLC-MS/MS, SCIEX-QTRAP 5500 tandem quadrupole mass spectrometry, SCIEX, United States), following the methodology outlined in Zhao et al. (2021) and Ge et al. (2024): the column temperature was maintained at 40°C; the mobile phase was composed of 0.1% formic acid (Fisher, United States) as eluent A and 0.1% acetonitrile (Fisher, United States) as eluent B; the flow rate was established at a constant value of 0.3 mL/min; the volume of injection was 5.00 μL. The linear gradient elution program was as follows: from 0 to 3 min, the concentration of solution B increased linearly from 15 to 35%; from 3 to 5 min, the concentration of solution B increased linearly from 35 to 45%; from 5 to 7 min, it increased linearly from 45 to 50%; linear equilibrium was maintained during the period from 7 to 7.5 min. The mass spectrometer was operated in positive ionization mode (ESI^+^) using an electrospray source. The parameters for the analysis of the mycotoxin, including *m*/*z* values, collision energy of parent ions, primary daughter ions, and selection criteria, are presented in Supplementary Table S1.

The analytical method for mycotoxin detection was validated according to the method described by Ge et al. (2024), and the standard curve was established. The signal-to-noise ratio S/N ≥3 was used as the limit of detection (LOD), and S/N ≥10 was used as the limit of quantification (LOQ). The method’s repeatability, instrument’s precision and stability were assessed by measuring the mycotoxin content, the peak area of six consecutive injections, and the relative standard deviation of the mycotoxin content detected at 0, 2, 4, 6, 12, and 24 h. The method accuracy was evaluated by conducting sample recovery experiments to determine the recovery rate and relative standard deviation of each mycotoxin.

### 2.2 Microbiome sequencing and analysis

The extraction and amplification of fungal DNA from *Radix Dipsaci* were conducted in accordance with the methods detailed in a previous report (He et al., 2023). The amplification of the ITS regions of the fungal 18S rRNA genes utilized primers ITS1F (5′-CTTGGTCATTTAGAGGAAGTAA-3′) and ITS2R (5′-GCTGCGTTCTTCATCGATGC-3′). Fungal PCR was conducted following the protocol in the previous study (He et al., 2023). The PCR products were detected through 2% agarose gel electrophoresis. Purified amplicons were pooled in equimolar ratios and paired-end sequenced on an Illumina MiSeq platform (Illumina, San Diego, United States) according to the standard protocols described by Majorbio Bio-Pharm Technology Co., Ltd. (Shanghai, China). The raw sequencing reads have been deposited into the NCBI Sequence Read Archive (BioProject ID PRJNA1103735).

Sequences in the library were clustered using USearch, operational taxonomic units (OTUs) were clustered at 97% similarity for non-repetitive sequences (excluding individual sequences), and chimeras were eliminated during clustering to derive representative sequences of OTUs. Utilizing the OTUs information, rarefaction curves and alpha diversity indices including observed OTUs, Ace, Chao richness and Shannon and Simpson index were calculated with Mothur v1.30.1. The similarity among the microbial communities in different samples was assessed through principal coordinate analysis principal coordinate analysis (PCoA) based on Bray–Curtis dissimilarity using Vegan v2.5-3 package.

### 2.3 Isolation and identification of potentially toxigenic fungi

#### 2.3.1 Isolation of potentially toxigenic fungi

Accurately weighing 15 g of the *Radix Dipsaci* contaminated with mycotoxins using an analytical balance into a conical bottle of 135 mL sterile water. The *Radix Dipsaci* sample powder was thoroughly dispersed by shaking for 20 min in a shaking incubator (GZX-400EF, Tianjin Test Instrument Co., Ltd., China). Serial dilutions ranging from 10^−1^ to 10^−4^ were meticulously prepared from the samples, following the documented procedure in Su et al. (2018). The prepared dilutions were inoculated on Potato Dextrose Agar (PDA) media, which consisted of 200 g of potato, 20 g of glucose (Tianjin Yongda Chemical Reagent Co., Ltd., China), 15 g of agar (Soleibao Biotechnology Co., Ltd., China), and 0.1 g of kanamycin (Soleibao Biotechnology Co., Ltd., China) dissolved in 1 L of water (Wei et al., 2023). Subsequently, the samples were incubated at a constant temperature of 28°C for a duration ranging from 5 to 7 days, throughout which the morphology of the colonies was meticulously observed and documented (Chen et al., 2020). Pure cultures were successfully isolated after multiple rounds of purification, and these pure cultures were then stored at a refrigerated temperature of 4°C.

#### 2.3.2 Morphological and molecular identification of potentially toxigenic fungi

Macroscopic characteristics, such as the growth rate, morphological alterations of mycelium, and the texture and coloration of purified fungal colonies, were systematically observed with the naked eye. The microscopic characteristics, such as conidia and conidiophores of the toxigenic fungi, were observed using a microscope (BX-41, Olympus Corporation Limited, Japan).

Fresh mycelia were collected from PDA surfaces and fungal DNA extraction was carried out following the protocol described in He et al. (2023). Amplification of the ITS2 region within the fungal DNA gene was achieved using primers ITS1 (5′-TCCGTAGGAACCTGCGG-3′) and ITS4 (5′-TCCTCCGCTTATTGATATGC-3′). PCR amplifications were carried out in a 20 μL reaction volume using the C1000Touch™ Thermal Cycler (Bio-Rad, United States). The system comprised 0.4 μL of each forward primer (ITS1) and reverse primer (ITS4), 2.0 μL of DNA template, 0.2 μL of EasyTaq DNA polymerase, 2.0 μL of 10 × EasyTaq buffer, 1.5 μL of 2.5 mM dNTPs, and 13.5 μL of H_2_O. The thermocycler was programmed for 35 cycles: denaturation at 95°C for 30 s, annealing at 58°C for 40 s, elongation at 72°C for 1 min, and final elongation at 72°C for 10 min. The PCR products were detected using the ChemiDoc^TM^ Touch Imaging System (Bio-Rad, United States) for digital imaging gel analysis. Following the PCR amplification, samples were sent to Shanghai Shenggong Biological Company (Shanghai, China) for sequencing. The resulting sequences were subjected to analysis via the BLAST program^1^ hosted on the NCBI website, to identify the genera and species of the fungal isolates. Then, the toxigenic fungi were subsequently subjected to phylogenetic analysis using reference sequences derived from ITS rDNA and closely related taxa available in GenBank. The fungus *Saccharomyces cerevisiae* was used as an outgroup for phylogenetic analyses. The phylogenetic analysis of sequences retrieved from GenBank, and the construction of phylogenetic trees were conducted using the MEGA 5.0 software. The evolutionary history was inferred using the neighbor-joining method, and the p-distance method was employed to calculate the evolutionary distance. The robustness of the branches was assessed through 1,000 bootstrap replications.

### 2.4 Analysis of key gene expression in mycotoxin synthesis

DNA extraction from fungal samples was conducted according to the protocol in He et al. (2023). The conserved regions of AFs, OTA, T-2, and ZEN were selected for the design of specific primers targeting their synthesis key genes *Aflr*, *PKS*, *Tri7*, and *PKS14*. PCR primers are detailed in Supplementary Table S2. The extracted fungal DNA was utilized as templates for PCR reactions to detect the presence of *Aflr*, *PKS*, *Tri7*, and *PKS14* in purified toxigenic fungi. The PCR reaction was conducted using a 20 μL reaction system, and the amplification procedure is detailed in section 2.3.2.

### 2.5 Verification of mycotoxins production

The isolated toxigenic fungi were introduced onto a substrate composed of irradiated sterilized rice and *Radix Dipsaci*, with the moisture level adjusted to 30%, followed by incubation at a temperature of 25°C for a duration of 14 days. After incubation, all cultures were dehydrated at 45°C and ground into a fine powder. Accurately weigh 2 g of powder using an analytical balance into a centrifuge tube. The centrifugal tube was supplemented with 10.0 mL of carbinol (lot No. 200343, Fresher, United States) containing 70% concentration. After thorough oscillation and centrifugation, 5 mL of the supernatant was transferred to a 10 mL volumetric flask. The sample, which was filtered using a 0.22 μm polytetrafluoroethylene-ethylene filter, was injected into the HPLC-MS/MS system for analysis.

### 2.6 Statistical analysis

Statistical analysis of the data was performed with GraphPad Prism 8.0 (GraphPad, La Jolla, CA, United States). Results were presented as mean ± SEM. Group differences were evaluated for significance with one-way ANOVA for normally distributed data, as determined by the Kolmogorov–Smirnov test, or with the Kruskal–Wallis test for non-normally distributed data. Significance was defined as *p* < 0.05. The results of statistical analysis are shown in Supplementary Tables S3, S4.

## 3 Result

### 3.1 Occurrence of mycotoxins in *Radix Dipsaci*

**Figure 1:**
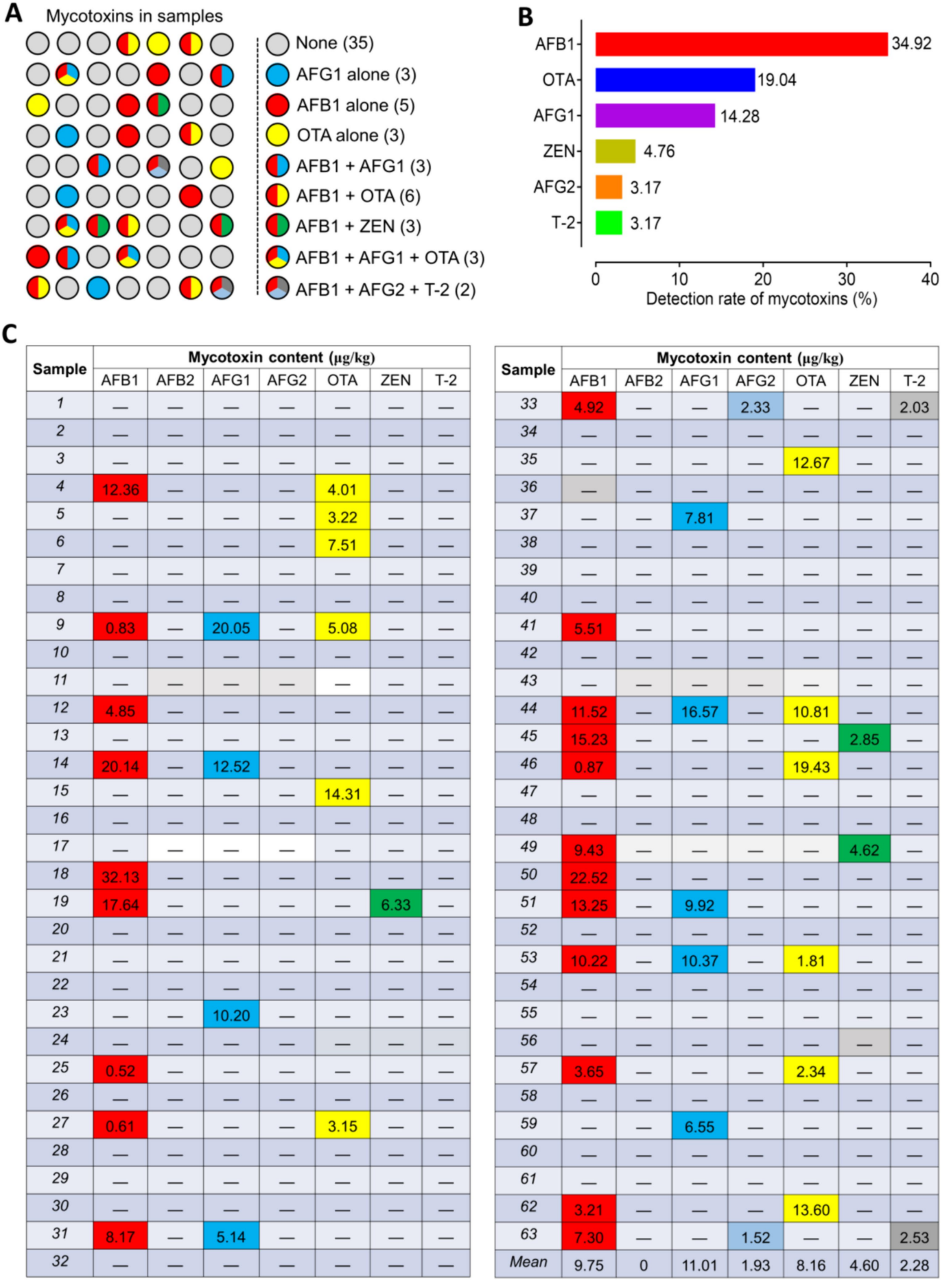
Occurrence of mycotoxins in *Radix Dipsaci*. **(A)** Determination of mycotoxins in 63 batches of *Radix Dipsaci* from major herbal medicine markets in five parts of China. None, samples were not contaminated with any mycotoxins; AFG_1_ alone, samples contaminated only with aflatoxin G_1_ (AFG_1_); AFB_1_ alone, samples contaminated only with aflatoxin B_1_ (AFB_1_); OTA alone, samples contaminated only with ochratoxin A (OTA); AFB_1_ + AFG_1_, samples contaminated with both AFB_1_ and AFG_1_; AFB_1_ + OTA, samples contaminated with both AFB_1_ and OTA; AFB_1_ + ZEN, samples contaminated with both AFB_1_ and zearalenone (ZEN); AFB_1_ + AFG_1_ + OTA, samples contaminated with AFB_1_, AFG_1_ and OTA; AFB_1_ + AFG_2_ + T-2, samples contaminated with both AFB_1_, aflatoxin G_2_ (AFG_2_) and T-2 toxin (T-2). **(B)** Percentages of samples contaminated with AFB_1_, OTA, AFG_1_, ZEN, AFG_2_ and T-2. **(C)** Levels of mycotoxins contamination of 63 batches *Radix Dipsaci* samples.

The UPLC-MS/MS method was utilized to detect mycotoxins in the *Radix Dipsaci* samples, revealing that out of the 63 batches samples, mycotoxins were identified in 28 batches, indicating a contamination rate of 44.4%. Among them, 11 batches were found to be contaminated with a single mycotoxin (AFB_1_, AFG_1_, OTA), while 17 batches were discovered to be contaminated with two or more mycotoxins (Figure 1A). The most prevalent mycotoxins detected in the positive samples were AFB_1_ (34.92%), OTA (19.04%), and AFG_1_ (14.28%) (Figure 1B). The contamination levels of AFB_1_, AFG_1_, AFG_2_, and OTA in the positive samples ranged from 0.52 to 32.13 μg/kg, 5.14 to 20.05 μg/kg, 1.52 to 2.33 μg/kg, and 1.81 to 19.43 μg/kg respectively, while the concentrations of ZEN and T-2 were found to range from 2.85 to 6.33 μg/kg and from 2.03 to 2.53 μg/kg, respectively. Among the samples contaminated with mycotoxins, 13 batches samples exhibited AFB_1_ levels exceeding 5 μg/kg, while in 5 batches samples OTA levels surpassed 10 μg/kg. Moreover, in 3 batches samples both AFB_1_ and AFG_1_ exceeded the threshold of 10 μg/kg. The ZEN contamination level in the sample was below 500 μg/kg, which meets the requirements of the Chinese pharmacopoeia (2020 edition) (Figure 1C). The findings imply that the *Radix Dipsaci* is prone to contamination by diverse mycotoxins.

### 3.2 Fungal diversity and community were altered in the *Radix Dipsaci* contaminated with different mycotoxins

**Figure 2:**
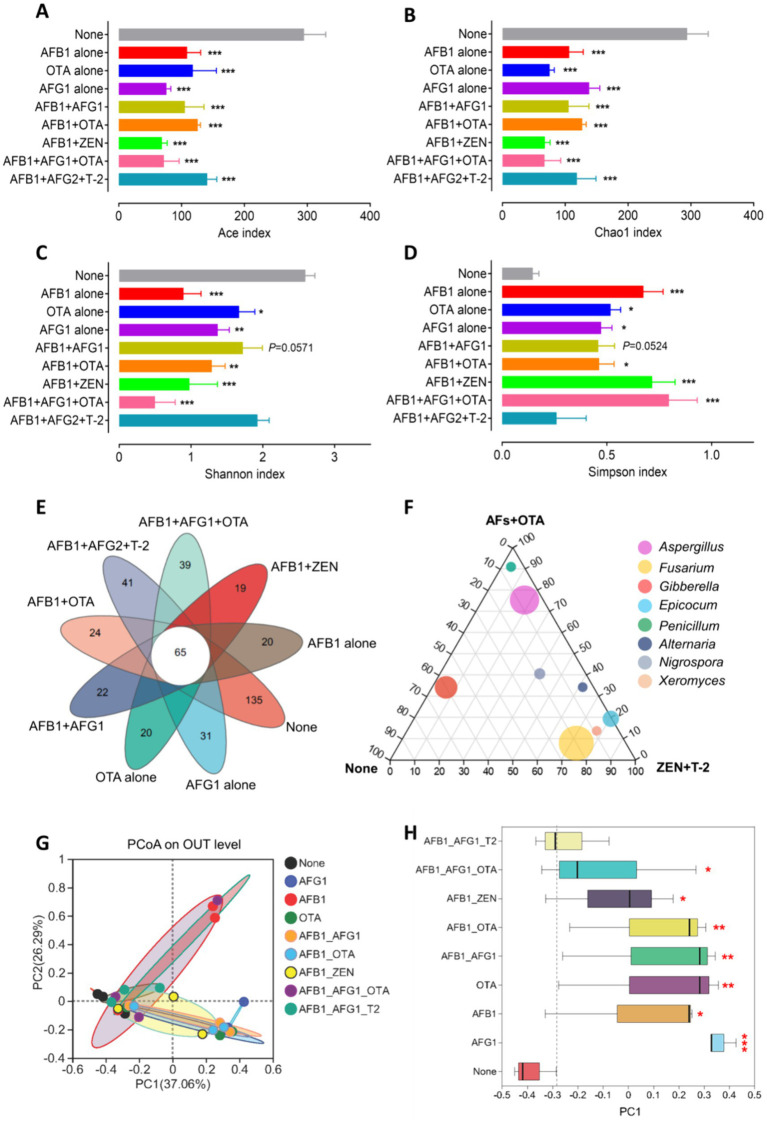
Fungal diversities were altered in the *Radix Dipsaci* contaminated mycotoxins. **(A–D)** Alpha diversity at the level of OTUs in fungal communities in *Radix Dipsaci* samples not contaminated with mycotoxins or contaminated with different mycotoxins. Microbial diversity was quantified using Chao’s, Ace’s, Shannon’s and Simpson’s diversity indices (*n* = 3). **(E)** Wayne’s analysis showed the changes in the number of operational taxonomic unit (OTU) of fungi in *Radix Dipsaci* samples contaminated with different mycotoxins. **(F)** The difference of fungal genus among none, AFs + OTA, and ZEN + T-2 evaluated using the two-tailed Wilcoxon test. **(G,H)** Principal component analysis (PCoA) of beta diversity in fungal communities in *Radix Dipsaci* samples not contaminated with mycotoxins or contaminated with different mycotoxins, based on Bray–Curtis distances. Data are mean ± SEM (*n* = 3). ^*^p < 0.05, ^**^p < 0.01, and ^***^p < 0.001 compared with none group by one-way ANOVA with Tukey’s multiple comparison *post hoc* test.

We prepared *Radix Dipsaci* samples that were not contaminated with mycotoxins or contaminated with different mycotoxins for conducting microbiome sequencing. Fungal alpha diversity was assessed through computation of Chao, Ace, Shannon, and Simpson indices at the operational taxonomic unit (OTU) level. The findings indicated a marked reduction in Ace and Chao indices of fungi in *Radix Dipsaci* contaminated with AFB_1_ alone, AFG_1_ alone, OTA alone, AFB_1_ + OTA, AFB_1_ + ZEN, or AFB_1_ + AFG_1_ + OTA, compared to non-contaminated samples, (*p* < 0.001) (Figures 2A,B), indicating that mycotoxins contamination reduced the richness of fungi in *Radix Dipsaci*. A notable decline in the Shannon index and a substantial rise in the Simpson index of fungi were observed in *Radix Dipsaci* contaminated with AFB_1_ alone, AFG_1_ alone, OTA alone, AFB_1_ + OTA, AFB_1_ + ZEN, or AFB_1_ + AFG_1_ + OTA, in contrast to non-contaminated samples, (*p* < 0.05) (Figures 2C,D), indicating that mycotoxins contamination reduced fungal diversity in *Radix Dipsaci*. We observed a lower number of fungal OTUs in all samples contaminated with mycotoxins when compared to those of the samples not contaminated with mycotoxins (Figure 2E). Additionally, we identified 65 OTUs common to all *Radix Dipsaci* samples and 20, 31, 20, 22, 24, 19, 39 and 41unique OTUs in samples contaminated with AFB_1_ alone, AFG_1_ alone, OTA alone, AFB_1_ + AFG_1_, AFB_1_ + OTA, AFB_1_ + ZEN, AFB_1_ + AFG_1_ + OTA and AFB_1_ + AFG_2_ + T-2 respectively (Figure 2E). We also found that the dominant fungi in samples not contaminated with mycotoxins were fungi of the genus *Gibberella*, the dominant fungi in samples contaminated with AFs and OTA were fungi of the genus *Aspergillus*, the dominant fungi in samples contaminated with ZEN and T-2 were fungi of the genus *Fusarium* (Figure 2F).

**Figure 3:**
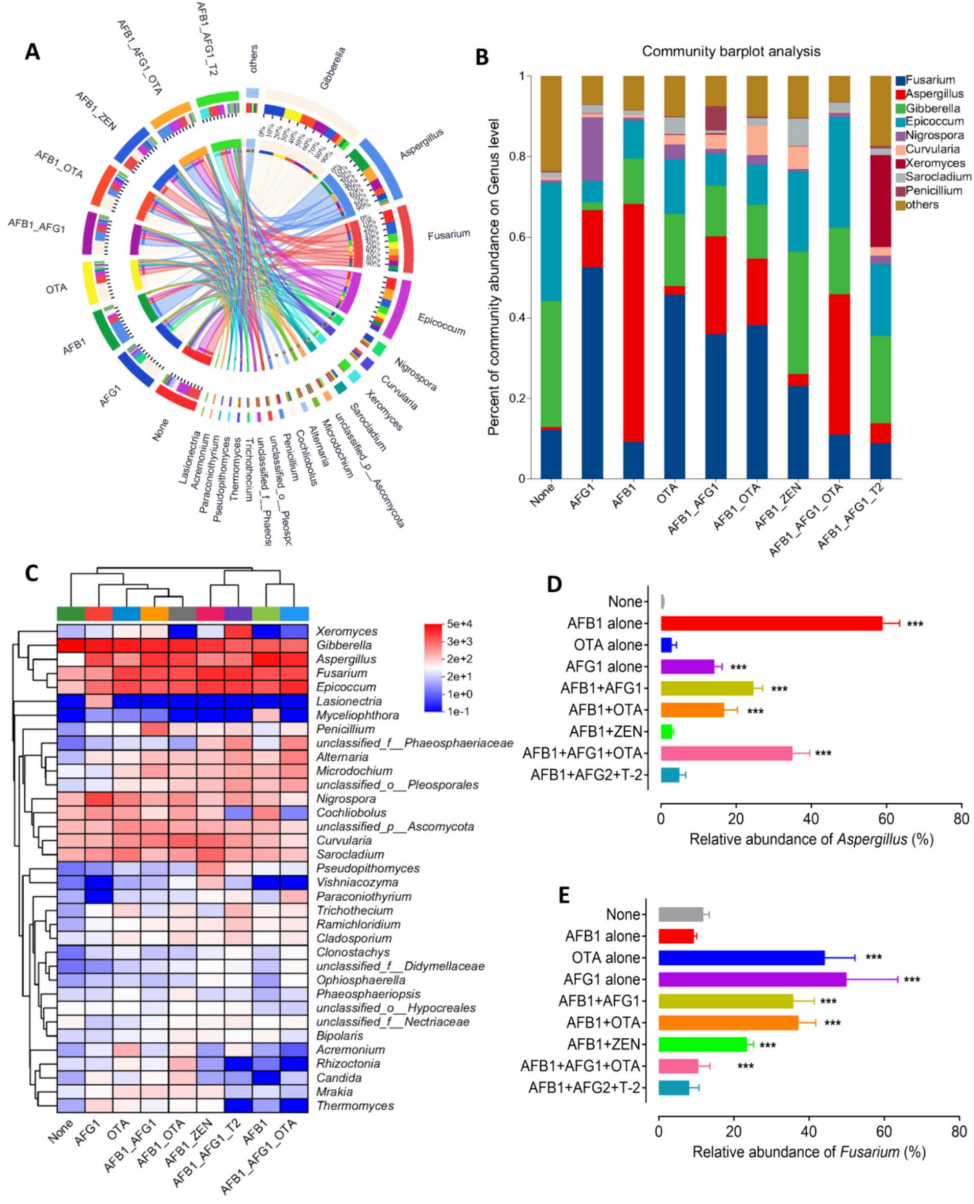
Fungal communities were altered in the *Radix Dipsaci* contaminated mycotoxins. **(A,B)** Relative abundances of fungal genus in *Radix Dipsaci* samples not contaminated or contaminated with different mycotoxins. **(C)** The difference of fungal genus among *Radix Dipsaci* samples not contaminated or contaminated with different mycotoxins. **(D,E)** Relative abundances of *Aspergillus* spp. and *Fusarium* spp. in *Radix Dipsaci* samples not contaminated or contaminated with different mycotoxins. Data are mean ± SEM (*n* = 3). ^***^p < 0.001 compared with none group by one-way ANOVA with Tukey’s multiple comparison *post hoc* test.

Beta diversity was analyzed through principal coordinate analysis (PCoA) plots using nonphylogenetic Bray–Curtis metrics to assess differences in microbial composition (OTU) among these groups (Figure 2G). A distinct segregation was evident between the samples contaminated with AFB_1_ alone, AFG_1_ alone, OTA alone, AFB_1_ + AFG_1_, AFB_1_ + OTA, AFB_1_ + ZEN, and AFB_1_ + AFG_1_ + OTA, and the mycotoxin-free samples along the primary axis of the first principal component (PC1), *p* < 0.05 (Figure 2H), indicating that contamination with mycotoxins can alter the composition of the fungal community. For instance, at the genus level, a notable rise in the prevalence of *Aspergillus* fungi was observed in samples contaminated solely with AFB_1_, AFG_1_, AFB_1_ + AFG_1_, AFB_1_ + OTA, and AFB_1_ + AFG_1_ + OTA. Conversely, there was a significant surge in the abundance of *Fusarium* fungi in samples contaminated with OTA alone, AFG_1_ alone, AFB_1_ + AFG_1_, and AFB_1_ + OTA, (*p* < 0.001) (Figures 3A–E). These results indicate that fungal diversity and community were altered, and the abundance of *Aspergillus* and *Fusarium* were increased in the *Radix Dipsaci* contaminated with different mycotoxins.

### 3.3 Isolation and identification of potentially toxigenic fungi in *Radix Dipsaci*

**Figure 4:**
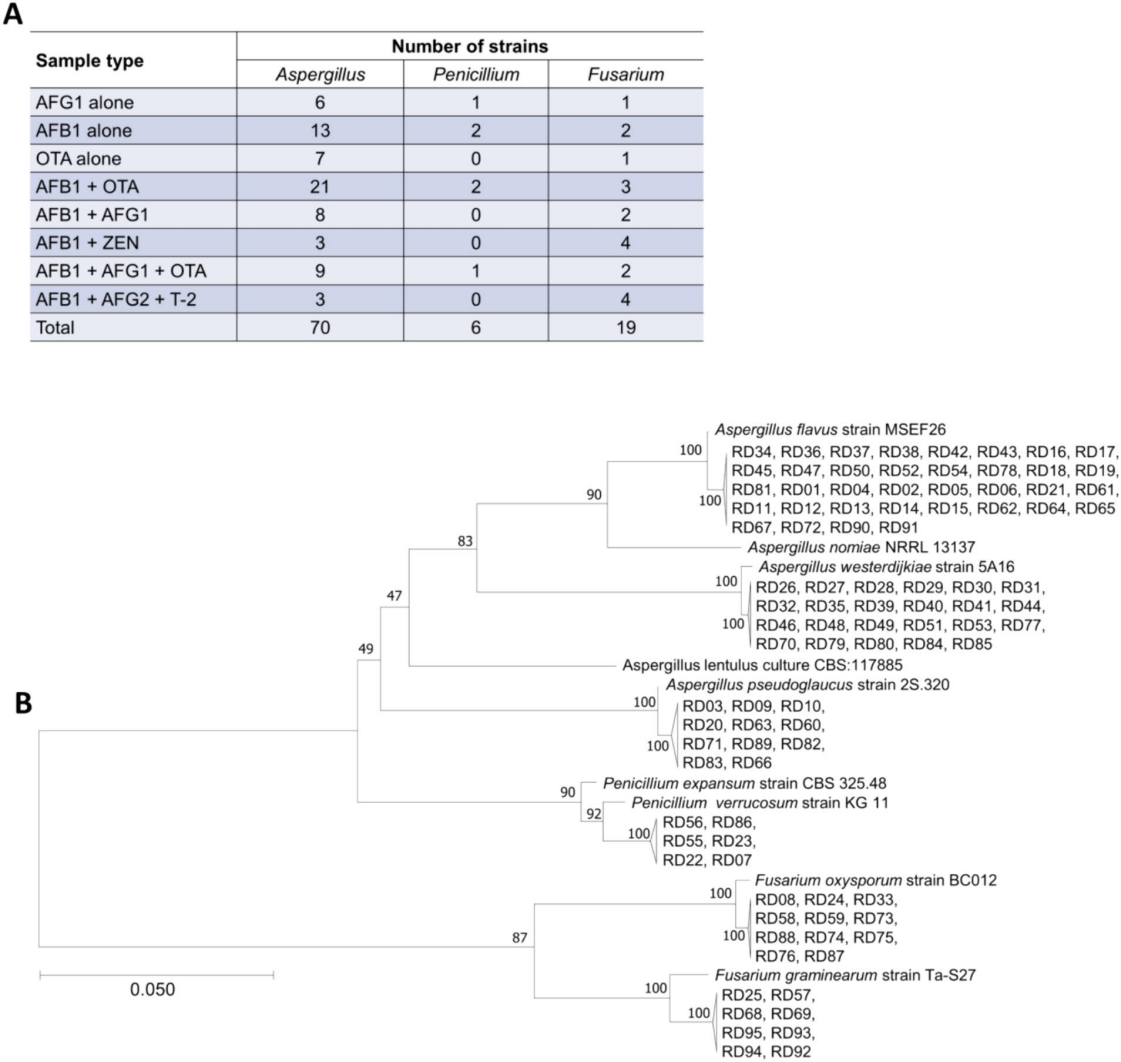
Isolation and molecular identification of potentially toxigenic fungi from *Radix Dipsaci* contaminated with mycotoxins. **(A)** The quantities of *Aspergillus* spp., *Penicillium* spp. and *Fusarium* spp. isolated from *Radix Dipsaci* sample contaminated with various mycotoxins. **(B)** Molecular identification of potentially toxigenic fungi from *Radix Dipsaci* contaminated with mycotoxins. Phylogenetic tree based on gene sequences of specific fungi, inferred by using the maximum likelihood method, on 36 *A. flavus*, 23 *A. westerdijkiae*, 11 *A. pseudoglaucus*, 11 *F. oxysporum*, eight *F. graminearum* and six *P. verrucosum* isolated from *Radix Dipsaci*, compared to reference sequences for corresponding species.

Ninety-five strains of toxigenic fungi were isolated from 28 batches of *Radix Dipsaci* contaminated with various mycotoxins. A greater number of *Aspergillus* strains were isolated from *Radix Dipsaci* samples contaminated with AFG_1_, AFB_1_, OTA, AFB_1_ + OTA, AFB_1_ + AFG_1_ and AFB_1_ + AFG_1_ + OTA, resulting in a total of 64 *Aspergillus* species being identified. More *Fusarium* strains were isolated from the *Radix Dipsaci* samples contaminated with AFB_1_ + ZEN and AFB_1_ + AFG2 + T-2, resulting in a total of 8 *Fusarium* species (Figure 4A).

Through the phylogenetic tree indicated that 36 potentially toxigenic fungal strains shared a close genetic relationship with *A. flavus* strain MSEF26, showing a 100% sequence homology. The 23 toxigenic fungal strains showed a 100% similarity with *A. westerdijkiae* strain 5A16. The homology between the six toxigenic fungal strains and *Penicillium verrucosum* strain KG11 was determined to be 92%. The 11 potentially toxigenic fungal strains exhibited a 100% homology with *F. oxysporum* strain BC012. The eight toxigenic fungal strains demonstrated a 100% similarity with *F. graminearum* strain Ta-S27 (Figure 4B).

The purified strains were observed for pigment and morphological characteristics (as showed Supplementary Figure S1). The colonies of *P. verrucosum* showed displaying green coloration, white margins, and beige undersides. The conidia exhibited displaying a spherical to subspherical shape. The colony of *A. flavus* showed featuring a loosely structured pale-yellow surface, the mycelium exhibited accompanied by flask-shaped at the apex. The colony of *A. westerdijkiae* showed velvety texture. The apical capsule of the conidium was spherical, with the conidia also being spherical in shape. The colony of *Aspergillus pseudoglaucus* exhibited rapid growth on PDA and was floccose, flat, brilliant greenish yellow. The reverse side of the colony appeared pale yellow to brilliant yellow. The colony of *F. graminearum* diffuse aerial mycelium that was white on the front but had reddish pigmentation on the reverse side of the culture plate. The *F. oxysporum* colony was white to lavender, with red pigment precipitation from the back side of the plate. Both large and small conidia were observed in the colonies. The small conidia were ovoid. The large conidia were sickle - shaped.

**Figure 5:**
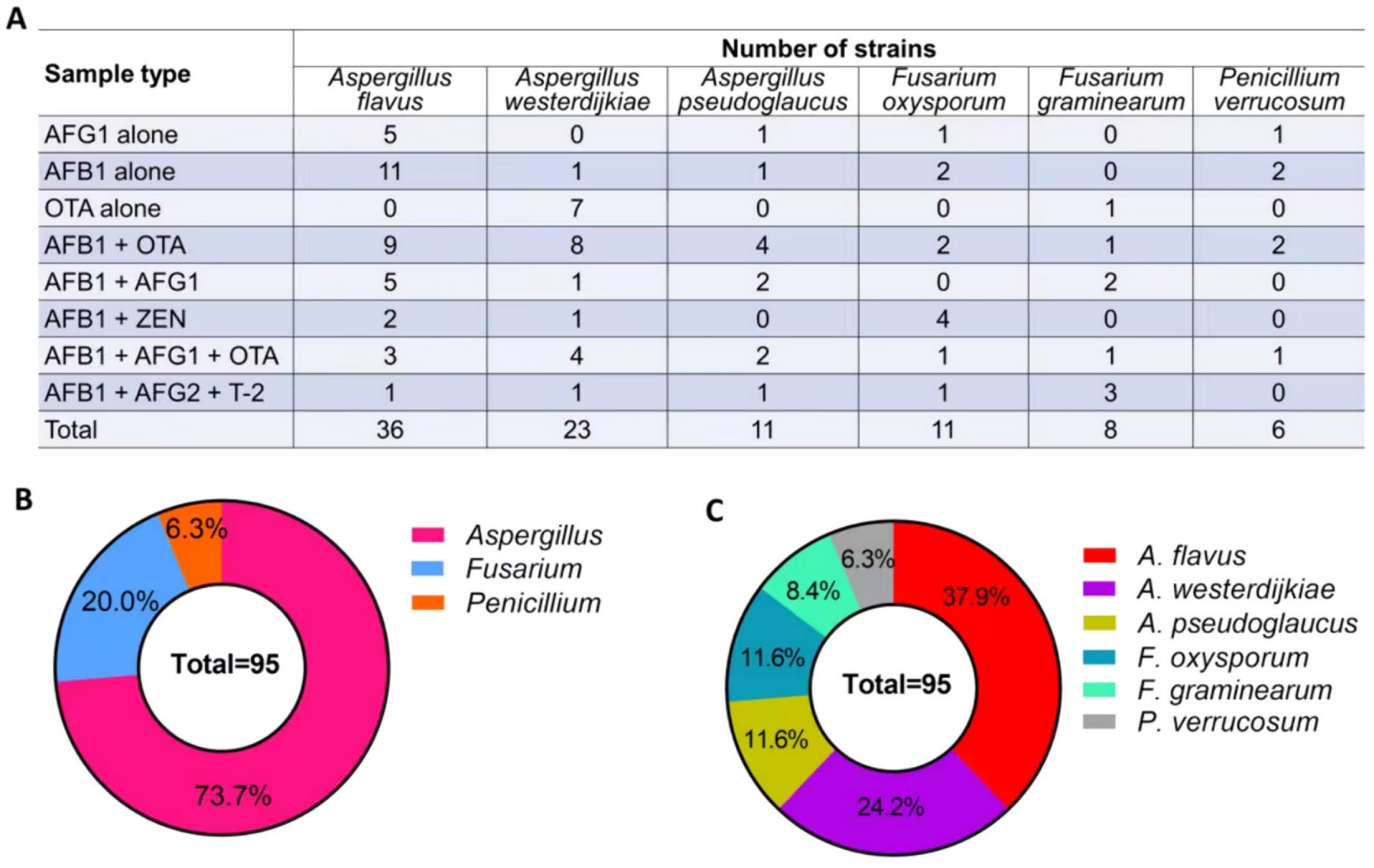
Quantity and frequency of potentially toxigenic fungi in *Radix Dipsaci*. **(A)** The quantity of potentially toxigenic fungi isolated from *Radix Dipsaci* contaminated with mycotoxins. **(B,C)** The frequency of potentially toxigenic fungi isolated from *Radix Dipsaci* contaminated with mycotoxins.

The results revealed that *A. flavus* exhibited the highest frequency of isolation from the samples contaminated with AFG_1_ or AFB_1_. The predominant toxigenic fungi isolated from the samples contaminated with OTA was *A. westerdijkiae*. A higher frequency of occurrence of *A. flavus* and *A. westerdijkiae* was noted in samples contaminated with AFB_1_ and OTA. Moreover, the presence of *A. flavus*, *F. oxysporum*, and *F. graminearum* was observed more frequently in samples contaminated with AFB_1_ and ZEN or T-2 (Figure 5A).

A total of 95 strains of fungi were isolated from the *Radix Dipsaci* samples contaminated with mycotoxins, predominantly comprising *Aspergillus* (73.7%), *Fusarium* (20.0%), and *Penicillium* (6.3%) (Figure 5B). Subsequent investigations unveiled that a total of 36 strains of *A. flavus* were isolated from the contaminated samples, constituting approximately 37.9% of the overall strain population. Twenty-three strains of *A. westerdijkiae* were isolated from the contaminated samples, accounting for 24.2%. *A. pseudoglaucus* and *F. oxysporum* strains were evenly distributed, with each species comprising 11 strains, accounting for 11.6% respectively. The presence of eight strains of *F. graminearum* accounted for 8.4%, while the presence of six strains of *P. verrucosum* accounted for 6.3% (Figure 5C).

### 3.4 Identification of key genes for mycotoxins biosynthesis of toxigenic fungi in *Radix Dipsaci*

**Figure 6:**
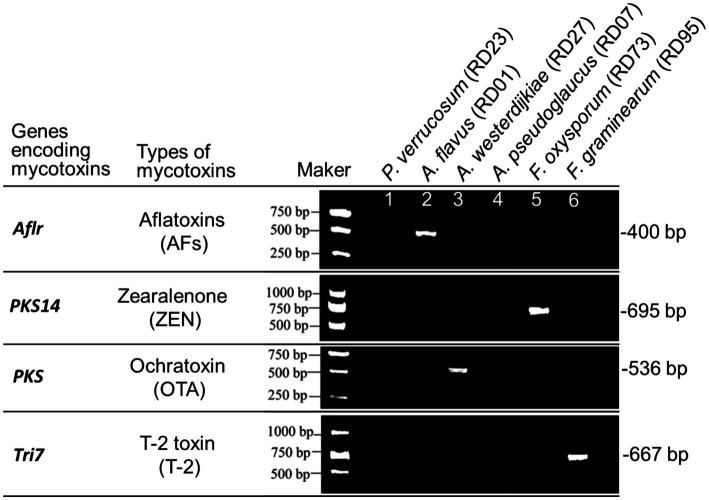
PCR identification of key genes for mycotoxins biosynthesis in potentially toxigenic fungi from *Radix Dipsaci*.

The pivotal genes *Aflr*, *PKS*, *Tri7*, and *PKS14* associated with the biosynthesis of AFs, OTA, T-2, and ZEN were identified through PCR amplification. The results indicated the presence of the *Aflr* gene in *A. flavus*, implying its capability for AFs production. The detection of the *PKS14* gene in *F. oxysporum* implies the potential for ZEN production by this fungus. The identification of the *PKS* gene in *A. westerdijkiae* indicates the potential for OTA production by this fungus. The presence of the *Tri7* gene in *F. graminearum* indicates its potential for T-2 biosynthesis (Figure 6).

### 3.5 Verification of mycotoxins production for toxigenic fungi in *Radix Dipsaci*

The production of mycotoxins by fungi expressing key genes involved in mycotoxin biosynthesis on rice and the *Radix Dipsaci* matrix was identified through the utilization of an immunoaffinity column coupled with HPLC-MS/MS (Supplementary Figure S2). The results uncovered that *A. flavus* simultaneously produced AFB_1_ and AFG_1_ in both the rice and *Radix Dipsaci* matrices. In the rice matrix, the levels of AFB_1_ and AFG_1_ produced were 0.61 ± 0.20 μg/kg and 0.89 ± 0.21 μg/kg, respectively, whereas in the *Radix Dipsaci* matrix, they were 0.18 ± 0.05 μg/kg and 0.13 ± 0.04 μg/kg, respectively. *A. westerdijkiae* demonstrated the ability to produce OTA in both the rice matrix and *Radix Dipsaci* matrix, with concentrations of 0.72 ± 0.16 μg/kg in the rice matrix and 0.06 ± 0.02 μg/kg in the *Radix Dipsaci* matrix. *F. oxysporum* exhibited the capacity to produce ZEN in both the rice matrix and *Radix Dipsaci* matrix, with ZEN contents of 4.02 ± 0.42 μg/kg in the rice matrix and 2.15 ± 0.36 μg/kg in the *Radix Dipsaci* matrix. The production of T-2 by *F. graminearum* in the rice matrix was below the limit of quantification, and no detectable levels of T-2 were observed in the *Radix Dipsaci* matrix (Tables 1, 2).

**Table 1:** Amount of mycotoxins produced in rice (μg/kg).

Toxigenic fungus	AFB_1_	AFG_2_	OTA	ZEN	T-2
*A. flavus*	0.61 ± 0.20	0.89 ± 0.21	0	0	0
*A. westerdijkiae*	0	0	0.72 ± 0.16	0	0
*F. oxysporum*	0	0	0	4.02 ± 0.42	0
*F. graminearum*	0	0	0	0	<LOQ

**Table 2:** Amount of mycotoxins produced in *Radix Dipsaci* (μg/kg).

Toxigenic fungus	AFB_1_	AFG_2_	OTA	ZEN	T-2
*A. flavus*	0.18 ± 0.05	0.13 ± 0.04	0	0	0
*A. westerdijkiae*	0	0	0.06 ± 0.02	0	0
*F. oxysporum*	0	0	0	2.15 ± 0.36	0
*F. graminearum*	0	0	0	0	<LOQ

## 4 Discussion

Contamination of Chinese herbal medicine with mycotoxins can reduce or even lose its therapeutic effect, which is potentially harmful to human health. The current imperative in ensuring the quality and safety of Chinese herbal medicines lies in the meticulous identification of sources and circumstances surrounding mycotoxin contamination within these medicinal products. In the present study, we assessed the mycotoxins, mycobiota, and toxigenic fungi present in the traditional medicinal herb *Radix Dipsaci*. Our findings demonstrate that *Radix Dipsaci* is prone to contamination by a combination of AFB_1_, AFG_1_, AFG_2_, OTA, ZEN, and T-2. The fungal diversity and community were altered in the *Radix Dipsaci* contaminated with diverse mycotoxins. There was an increase in the abundance of *Aspergillus* and *Fusarium* in *Radix Dipsaci* contaminated with AFs and ZEN. Through the utilization of morphological identification, molecular identification, mycotoxin synthase gene identification, and toxin production verification, we validated that AFB_1_ and AFG_1_ mainly originated from *A. flavus*, OTA primarily originated from *A. westerdijkiae*, ZEN primarily originated from *F. oxysporum*, and T-2 primarily originated from *F. graminearum* in *Radix Dipsaci*. This study signifies a groundbreaking endeavor in the identification of mycotoxins and their toxigenic fungi in *Radix Dipsaci*, to the best of our knowledge.

Inadequate handling during the processing and storage of traditional Chinese herbal medicine may lead to contamination by toxigenic fungi, resulting in quality degradation and jeopardizing safe clinical usage (Chen et al., 2020). Studies have shown that root herbs are particularly vulnerable to mycotoxin contamination. Huang et al. reported that out of 31 batches of *Glycyrrhizae Radix* et *Rhizoma* samples, mycotoxin contamination was detected in 17 batches, resulting in a detection rate of 54.8% (Huang et al., 2018). In this study, out of 36 batches of *Radix Dipsaci* samples, 28 batches were found to be contaminated with toxigenic fungi, resulting in an overall contamination rate of 44.4%. The elevated mycotoxin contamination in *Radix Dipsaci* and *Glycyrrhizae Radix* et *Rhizoma* could be attributed to its classification as a root herb with an extended growth cycle typically exceeding 3 years, necessitating prolonged soil contact during development, thus facilitating the colonization of toxigenic fungi from the soil. Moreover, sweating during processing predisposes *Radix Dipsaci* to toxigenic fungal growth. The coarse exterior of the herb promotes the adhesion of fungal spores, heightening its vulnerability to contamination throughout storage and transit. Consequently, *Radix Dipsaci* is susceptible to potential toxigenic fungal contamination across the phases of cultivation, processing, storage, and transit.

The invasion of toxigenic fungi into medicinal materials results in the release of mycotoxins, which serve to inhibit the growth of competing fungi, thereby enabling the toxigenic fungi to access more nutrients. Consequently, the invasion of toxigenic fungi and the production of mycotoxins can disrupt the microbial community of the host (Pfliegler et al., 2019). Our investigation revealed a noteworthy reduction in the Ace, Chao, and Shannon indices of fungal diversity, alongside a marked increase in the Simpson index, within mycotoxin-contaminated *Radix Dipsaci*, in contrast to uncontaminated samples. These findings indicate a diminishment in fungal richness and diversity due to mycotoxin contamination in *Radix Dipsaci*. These results suggest that contamination by mycotoxins decreases the richness and diversity of fungi in *Radix Dipsaci*. The results of beta diversity analysis revealed a distinct separation between the samples contaminated with individual mycotoxins (AFB_1_, AFG_1_, OTA), combinations of mycotoxins (AFB_1_ + AFG_1_, AFB_1_ + OTA, AFB_1_ + ZEN, AFB_1_ + AFG_1_ + OTA), and the uncontaminated samples along the principal component axis, indicating that mycotoxin contamination has the potential to alter the fungal community structure. We also observed that the predominant fungi in samples contaminated with AFs and OTA were of the genus *Aspergillus*, a significant source of toxigenic fungi responsible for various AFs and ochratoxins (Jedidi et al., 2017). The dominant fungi in samples contaminated with ZEN and T-2 were fungi of the genus *Fusarium*, which belong to a major source of toxigenic fungus responsible for various mycotoxins including ZEN, T-2, and deoxynivalenol (Tima et al., 2016). We observed a significant increase in the abundance of *Aspergillus* and *Fusarium* in mycotoxin-contaminated *Radix Dipsaci*, indicating that the mycotoxins in *Radix Dipsaci* may originate from *Aspergillus* and *Fusarium* fungi.

Studies have shown that the most carcinogenic mycotoxins are AFs, encompassing AFB_1_, AFB_2_, AFG_1_, and AFG_2_ (Wong and Hsieh, 1976). The contamination of AFs is a prevalent issue in Chinese herbal medicines, with the direct contact between the roots and soil of root Chinese herbal medicines being particularly susceptible to AFs contamination. Han et al. (2010) detected 16 batches contaminated with AFs out of a total of 30 batches of Chinese medicinal herbs, which included *Puerariae Lobatae Radix*, leading to a detection rate of 53.3%. Liu et al. (2012) revealed that the incidence rates of AFB_1_, AFB_2_, AFG_1_, and AFG_2_ in traditional Chinese medicine samples contaminated with AFs were 15.52, 14.37, 6.32, and 2.30% respectively. In this study, *Radix Dipsaci* was identified as a type of herbal root with an observed occurrence rate of AFs at 52.37%. Among the positive samples, AFB_1_, AFG_1_ and AFG_2_ were detected at rates of 34.92, 14.28 and 3.17%, respectively. The results showed that rhizome herbs such as *Radix Dipsaci* and *Puerariae Lobatae Radix* were very sensitive to AFs, particularly AFB1. The nutrient-rich properties of root herbs, such as *Radix Dipsaci*, may create an optimal growth environment for toxigenic fungi, thereby facilitating their contamination by AFs. Scientific literature has documented at least 16 *Aspergillus* species capable of producing AFs (Frisvad et al., 2019). *A. flavus* and *A. parasiticus* are recognized as the primary producers of AFs (Abrar et al., 2013). In this study, *A. flavus* was also isolated from *Radix Dipsaci* contaminated with AFB_1_ or AFG_1_. *A. flavus* exhibited a high frequency of isolation and expressed *Aflr*, a pivotal synthetic gene responsible for aflatoxin synthesis. Furthermore, it was shown that *A. flavus* had the ability to consistently produce AFB_1_ and AFG_1_. The results suggest that *A. flavus* contamination is the main factor contributing to the presence of AFB_1_ and AFG_1_ in *Radix Dipsaci*.

The group of ochratoxins includes OTA, ochratoxin B, and ochratoxin C (Qileng et al., 2018). Among these, OTA is the most toxic, demonstrating nephrotoxic, hepatotoxic, teratogenic, neurotoxic, and carcinogenic effects in animals (Tarazona et al., 2018; Yu et al., 2018). Currently, the issue of OTA contamination in traditional Chinese herbal medicine is increasingly severe, garnering significant attention from relevant stakeholders in society. Trucksess et al. (2007) identified OTA contamination in 29 out of 39 batches of *Zingiberis Rhizoma Recens*, resulting in a positive sample incidence rate of 74.4%; among the 10 batches of *Ginseng Radix* et *Rhizoma*, OTA contamination was detected in four batches, with a detection rate of 40.0%. The results of this study revealed that the detection rate of OTA in *Radix Dipsaci*, a type of root herb, was 19.04%. The susceptibility of *Radix Dipsaci*, *Zingiberis Rhizoma Recens*, and *Ginseng Radix* et *Rhizoma* to OTA contamination may be attributed to the high protein, polysaccharide, and starch content in root herbs like *Radix Dipsaci*. These components serve as carbon sources for toxigenic fungi, facilitating their growth and proliferation, ultimately leading to OTA contamination. The fungi identified in relevant studies as producers of OTA include *A. westerdijkiae*, *A. steynii*, *A. niger*, and *P. verrucosum* (Bui-Klimke and Wu, 2015; Gil-Serna et al., 2015). *A. westerdijkiae* was isolated from *Radix Dipsaci* samples contaminated with OTA. Subsequent investigations revealed that *A. westerdijkiae* exhibited the expression of *PKS*, a crucial synthetic gene responsible for OTA synthesis, and demonstrated continuous production of OTA in rice and *Radix Dipsaci* matrices, respectively. The results indicate that the contamination of *A. westerdijkiae* is the primary contributing factor to the presence of OTA in *Radix Dipsaci*.

ZEN is widely distributed and shares structural similarities with endogenous estrogen, enabling it to competitively bind to estrogen receptors (Gao et al., 2018; Sun et al., 2022). This interaction disrupts hormone synthesis and metabolism, leading to aberrant estrogenic effects, damage to reproductive organs, and disturbances in reproductive hormone levels (Santos-Cruz et al., 2023; Takemura et al., 2007). Studies have found that agricultural products such as Chinese herbs are susceptible to ZEN contamination. Liao et al. discovered that 28.16% of the samples from 103 batches of Chinese medicinal herbs, including *Nelumbos Semen* and *Coicis Semen* were contaminated with ZEN. Notably, the ZEN content in five batches of *Coicis Semen* samples exceeded the maximum residue limit (Liao et al., 2023). The presence of ZEN in small amounts has been detected in samples of *Ginseng Radix* et *Rhizoma* and *Panacis Quinquefolii Radix*, and the crude extract of *Ginseng Radix* et *Rhizoma* roots in accordance with related studies (Gray et al., 2004; Trucksess and Scott, 2008). Our study revealed that only three out of the 36 batches of *Radix Dipsaci* tested positive for ZEN contamination, resulting in a low detection rate of 4.76%. The findings indicated that seed herbs, such as *Coicis Semen*, exhibited higher susceptibility to ZEN contamination compared to root herbs, such as *Radix Dipsaci*. The susceptibility of seed herbs to ZEN contamination may be attributed to their high content of rich fats and proteins. Currently, it has been demonstrated that ZEN is a toxic secondary metabolite generated by multiple strains of *Fusarium*, including *F. graminearum*, *F. verticillioides*, *F. oxysporum* and *F. acuminatum* (Ropejko and Twaruzek, 2021). *F. oxysporum* was isolated from *Radix Dipsaci* contaminated with ZEN. Subsequent investigations unveiled that *F. oxysporum* displayed the expression of *PKS14*, a key biosynthetic gene responsible for ZEN production, and demonstrated sustained synthesis of ZEN in both rice and *Radix Dipsaci* matrices. The findings suggest that the presence of ZEN in *Radix Dipsaci* is primarily attributed to contamination by *F. oxysporum*.

T-2 is the most toxic type A trichothecene mycotoxin, exhibiting cytotoxic, genotoxic, reproductive toxic and neurotoxic effects (Li et al., 2022; Yang et al., 2021). The contamination of T-2 was found to be particularly prevalent in traditional Chinese herbs. Boško et al. (2022) analyzed the contamination of *Silybi Fructus* samples with T-2, and it was found that all the examined samples were contaminated. Similarly, Wang et al. (2014) discovered that out of 17 batches of *Puerariae lobatae Radix* samples, two batches were contaminated with T-2, resulting in a contamination rate of 11.8%. The current study detected the existence of T-2 contamination in two batches of samples, with a positive sample incidence rate of 3.17%. The findings indicated that fruiting herbs exhibited a higher susceptibility to T-2 contamination, whereas root herbs demonstrated a lower susceptibility. The presence of fruit herbs may contribute to a more conducive growth environment for toxigenic fungi, thereby leading to their T-2 contamination. T-2 is a type A trichothecene toxin produced by various *Fusarium* species, including *F. graminearum*, *F. lateritium*, *F. oxysporum* and *F. tricinctum* (Rabie et al., 1986). *F. graminearum* was isolated from *Radix Dipsaci* samples contaminated with T-2, which exhibited the expression of *Tri7*, a crucial gene involved in T-2 synthesis, and demonstrated its capability to produce T-2 in rice matrix. The findings suggest that contamination by *F. graminearum* may the primary factor contributing to the presence of T-2 in *Radix Dipsaci*.

Chinese herbal medicines are susceptible to mycotoxins, the presence of a substantial quantity of fungal spores in the planting soil, combined with high temperature and humidity conditions, creates an optimal environment for fungal growth (Gonzalez-Curbelo and Kabak, 2023; Ahmad et al., 2022). The presence of excess water in herbal medicine can additionally facilitate the proliferation of fungi. Inadequate implementation of comprehensive quality control measures, detection methods, risk assessment, and monitoring systems are also contributing factors. The current primary objective is to effectively manage mycotoxins in Chinese herbs, such as *Radix Dipsaci*, to ensure the quality and safety of Chinese herbs. Therefore, it is crucial to employ rational pesticide and fertilizer usage, implement thorough disinfection and soil improvement measures prior to planting, maintain appropriate water content levels during storage, conduct regular quality testing of medicinal materials, promptly isolate and treat contaminated varieties, develop industry standards and practices for mycotoxins, establish comprehensive standards for monitoring and testing the quality of traditional Chinese medicine, as well as regularly assess and monitor the risk of mycotoxin contamination in order to effectively prevent mycotoxin.

## 5 Conclusion

The findings indicate that *Radix Dipsaci* is susceptible to contamination by a combination of aflatoxins, OTA, ZEN, and T-2. The detection rate of aflatoxin exceeded the standard limit in 20.63% of the samples, and the OTA toxin in 7.93% of the samples exceeded the limit standard. Neither ZEN nor T-2 exceeded the standard limit. Aflatoxins, OTA, ZEN, and T-2 primarily originate from *A. flavus*, *A. westerdijkiae*, *F. oxysporum* and *F. graminearum* in *Radix Dipsaci*. Mycotoxins contamination has been implicated in the alteration of fungal communities within *Radix Dipsaci*, especially the increase of the abundance of *Aspergillus* and *Fusarium*. To ensure the safety of *Radix Dipsaci*, future work is needed to monitor more mycotoxins, such as fumonisins and deoxynivalenol. Our findings presented in this study contribute significantly to a more comprehensive understanding of the causal agents responsible for mycotoxin contamination in *Radix Dipsaci* and their potential implications for consumer safety. The available data also offers valuable insights into the monitoring and control of other Chinese medicinal herbs that may potentially be contaminated by toxigenic fungi.

## Data Availability

The datasets presented in this study can be found in online repositories. The names of the repository/repositories and accession number(s) can be found in the article/Supplementary material.

## Footnotes

^1^ https://blast.ncbi.nlm.nih.gov/Blast.cgi
