# Supplementary material for: Evaluation of mycotoxins, mycobiota and toxigenic fungi in the traditional medicine Radix Dipsaci
Source: Front Microbiol. 2024 Sep 20;15:1454683. doi: 10.3389/fmicb.2024.1454683 (PMC11452847; doi:10.3389/fmicb.2024.1454683)
Supplement: Supplementary file 1 [file Data_Sheet_1.PDF]

Supplementary Figures and Tables

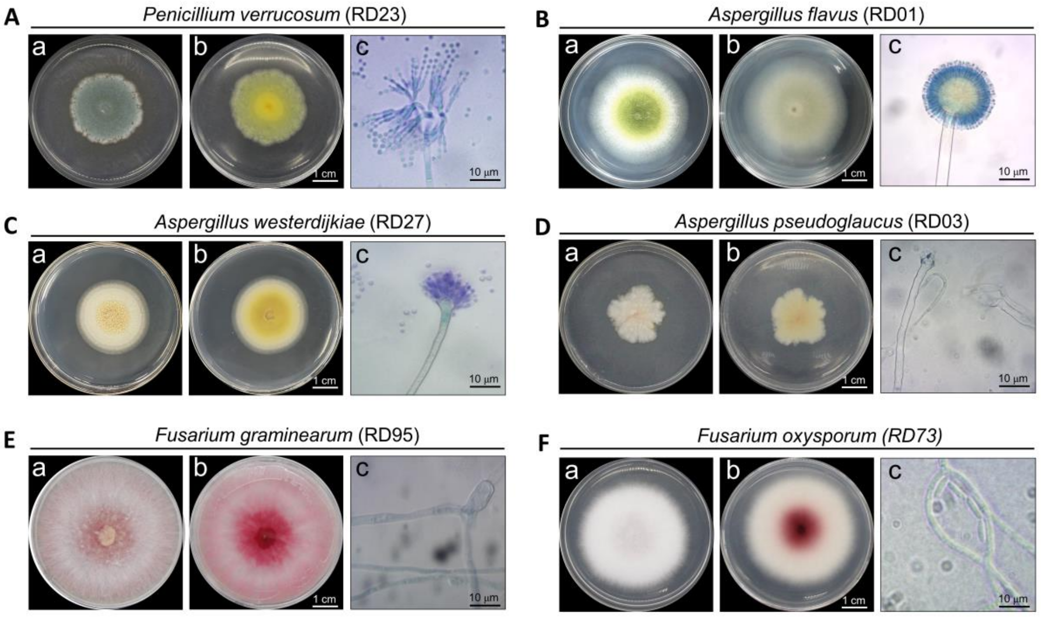

Supplementary Figure S1. Morphological characteristics of potentially toxigenic fungi from *Radix Dipsaci* contaminated with mycotoxins.

(A–F) Morphology and microstructure of *P. verrucosum, A. flavus, A. westerdijkiae, A. pseudoglaucus, F. graminearum* and *F. oxysporum.*

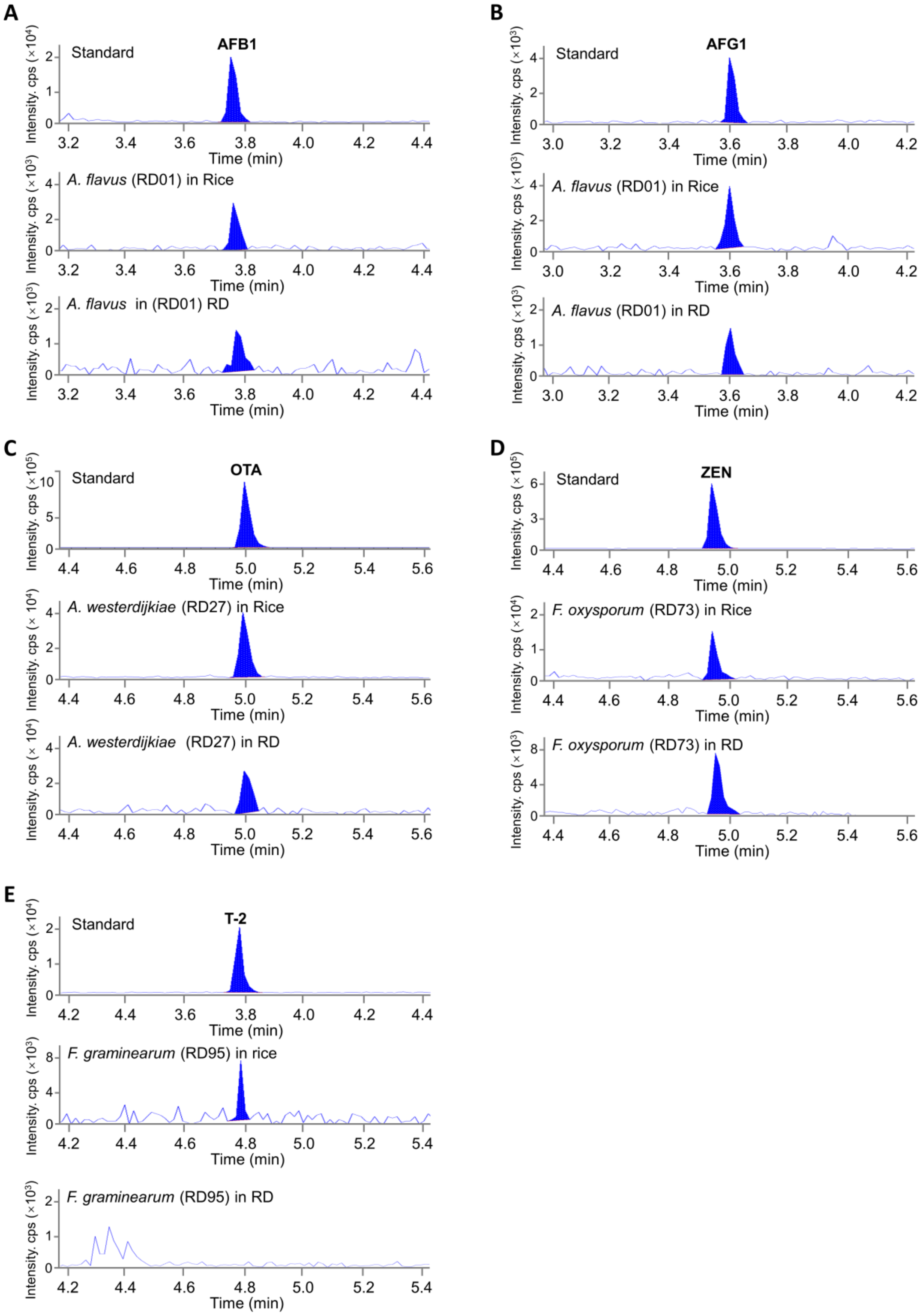

Supplementary Figure S2. Verification of mycotoxins production in potentially toxigenic fungi from *Radix Dipsaci*.

(A-E) The chromatogram of mycotoxins produced by toxigenic fungi in rice matrix and *Radix Dipsaci* matrix.

Supplementary Table S1 The parameters on the m/z and collision energy of parent ions, primary daughter ions, cone voltage and acquisition mode for mycotoxin

Mycotoxin	Parent ion (m/z)	Primary daughter ion (m/z)	cone voltage (eV)	Collision energy (eV)	Acquisition mode
AFB1	313.1	285.0[1)]/241.0[2)]	30	25/30	$[M+H]^{+}$
AFG1	329.1	243.1[1)]/283.1[2)]	30	25/20	$[M+H]^{+}$
OTA	404.1	238.9[1)]/301.0[2)]	20	15/25	$[M+H]^{+}$
T-2	489.0	245.0[1)]/327.0[2)]	22	28/22	$[M+Na]^{+}$
ZEN	319.1	283.0[1)]/301.0[2)]	10	15/10	$[M+H]^{+}$

Supplementary Table S2. Primer sets for key genes involved in mycotoxin synthesis.

Mycotoxin	Gene Target	Sequence (5’–3’)	Amplification Product (bp)
AFs	*Aflr*	GCACCCTGTCTTCCCTAACA<br>ACGACCATGCTCAGCAAGTA	400
OTA	*PKS*	GCCAGACCATCGACACTGCATGCTC<br>CGACTGGCGTTCCAGTACCATGAGC	536
T-2	*Tri7*	GCGAGGTATTGGAACRCCATG<br>TCTTCGATAATAATRCCGACAA	667
ZEN	*PKS14*	CCCTCGCCAAGCACCTCATC<br>AGTACCTTGCGAGCGACCTTC	695

Supplementary Table S3. The F value and P value in multiple comparisons of figure 2

Figure number	Marker	Groups	Mean Diff.	Type of ANOVA	95.00% CI of diff.	F, DFn, Dfd	P value
Figure 2A	Ace index	None vs AFB1 alone	-186.1	One-Way ANOVA Tukey’s post	-268.7 to -103.6	F (8, 18) = 17.20	<0.0001
		None vs OTA alone	-177.1		-259.6 to -94.57		<0.0001
		None vs AFG1 alone	-218.7		-301.3 to -136.2		<0.0001

		None vs AFB1+AFG1	-189.6	hoc tests	-272.2 to -107.1		<0.0001
		None vs AFB1+OTA	-169.2		-251.8 to -86.70		<0.0001
		None vs AFB1+ZEN	-226.3		-308.8 to -143.7		<0.0001
		None vs AFB1+AFG1+OTA	-222.9		-305.5 to -140.4		<0.0001
		None vs AFB1+AFG2+T-2	-153.9		-236.5 to -71.38		0.0001
Figure 2B	Chao 1 index	None vs AFB1 alone	-187.9	One-Way ANOVA Tukey's post hoc tests	-268.1 to -107.6	F (8, 18) = 18.42	<0.0001
		None vs OTA alone	-218.9		-299.2 to -138.6		<0.0001
		None vs AFG1 alone	-155.9		-236.1 to -75.61		<0.0001
		None vs AFB1+AFG1	-188.7		-269.0 to -108.5		<0.0001
		None vs AFB1+OTA	-167.0		-247.2 to -86.70		<0.0001
		None vs AFB1+ZEN	-226.7		-306.9 to -146.4		<0.0001
		None vs AFB1+AFG1+OTA	-227.2		-307.5 to -147.0		<0.0001
		None vs AFB1+AFG2+T-2	-175.3		-255.6 to -95.09		<0.0001
Figure 2C	Shannon index	None vs AFB1 alone	-1.696	One-Way ANOVA Tukey's post hoc tests	-2.758 to -0.6347	F (8, 18) = 8.492	0.0007
		None vs OTA alone	-0.9209		-1.982 to 0.1407-		0.1199
		None vs AFG1 alone	-1.219		-2.281 to -0.1579		0.0176
		None vs AFB1+AFG1	-0.8691		-1.931 to 0.1925		0.1621
		None vs AFB1+OTA	-1.302		-2.364 to -0.2408		0.0100
		None vs AFB1+ZEN	-1.614		-2.675 to -0.5521		0.0012
		None vs AFB1+AFG1+OTA	-2.092		-3.154 to -1.030		<0.0001
		None vs AFB1+AFG2+T-2	-0.6649		-1.726 to 0.3967		0.4479
Figure 2D	Simpson index	None vs AFB1 alone	0.5281	One-Way ANOVA Tukey's post hoc tests	0.1518 to 0.9043	F (8, 18) = 7.630	0.0028
		None vs OTA alone	0.3712		-0.005035 to 0.7474		0.0548
		None vs AFG1 alone	0.3256		-0.05064 to 0.7018		0.1215
		None vs AFB1+AFG1	0.3126		-0.06360 to 0.6889		0.1504
		None vs AFB1+OTA	0.3162		-0.06008 to 0.6924		0.1420
		None vs AFB1+ZEN	0.5697		0.1934 to 0.9459		0.0013
		None vs AFB1+AFG1+OTA	0.6497		0.2734 to 1.026		0.0003
		None vs AFB1+AFG2+T-2	0.1130		-0.2632 to 0.4892		0.9743
Figure 2H	PC1	None vs AFB1 alone	0.6537	One-Way	-0.0365 to 6544	F (8, 18) =	0.0352

		None vs OTA alone	0.7024	ANOVA Tukey's post hoc tests	0.1325to 0.6227	16.31	0.0054
		None vs AFG1 alone	0.7532		0.5486 to 0.7018		0.0008
		None vs AFB1+AFG1	0.7057		0.1098 to 0.6889		0.0013
		None vs AFB1+OTA	0.6813		0.1032 to 0.7325		0.0077
		None vs AFB1+ZEN	0.4038		-0.0876 to 0.9459		0.0232
		None vs AFB1+AFG1+OTA	0.2167		-0.1365 to 0.3654		0.0479
		None vs AFB1+AFG2+T-2	0.1135		-0.1632 to 0.2354		0.1362

Supplementary Table S4. The F value and P value in multiple comparisons of figure 3

Figure number	Marker	Groups	Mean Diff.	Type of ANOVA	95.00% CI of diff.	F, DFn, Dfd	P value
Figure 3 D	Relative abundance of Aspergillus (%)	None vs AFB1 alone	58.27	One-Way ANOVA Tukey's post hoc tests	50.38 to 66.17	F (8, 18) = 145.3	<0.0001
		None vs OTA alone	2.160		-5.738 to 10.06		0.9853
		None vs AFG1 alone	13.51		5.609 to 21.40		0.0003
		None vs AFB1+AFG1	23.89		16.00 to 31.79		<0.0001
		None vs AFB1+OTA	16.14		8.242 to 24.04		<0.0001
		None vs AFB1+ZEN	2.253		-5.644 to 10.15		0.9810
		None vs AFB1+AFG1+OTA	34.32		26.42 to 42.22		<0.0001
		None vs AFB1+AFG2+T-2	4.217		-3.681 to 12.11		0.6397
Figure 3 E	Relative abundance of Fusarium (%)	None vs AFB1 alone	-2.433	One-Way ANOVA Tukey's post hoc tests	-19.87 to 15.00	F (8, 18) = 21.93	0.9999
		None vs OTA alone	32.41		14.97 to 49.84		0.0001
		None vs AFG1 alone	38.16		20.73 to 55.60		<0.0001
		None vs AFB1+AFG1	24.02		6.583 to 41.45		0.0034
		None vs AFB1+OTA	25.39		7.959 to 42.83		0.0019
		None vs AFB1+ZEN	11.62		-5.817 to 29.05		0.3726
		None vs AFB1+AFG1+OTA	-1.310		-18.74 to 16.12		>0.9999
		None vs AFB1+AFG2+T-2	-3.697		-21.13 to 13.74		0.9972
